# Cases Showing the Effects of Suppressed Evacuations

**Published:** 1830-10-01

**Authors:** 


### LA PITIE.

### SOME CASES SHEWING THE EFFECTS OF THE SUPPRESSION OF HABITUAL EVACUATIONS.

Three or four cases are reported from the practice of M. Louis, in La Pitié, illustrative of the subject under consideration, which is by no means an uninteresting one.

*Case* 1. A voiturier, aged 25 years, had had an ulcer of long standing on each of his legs, of which he was cured in La Pitié in the space of six weeks, and was discharged. In about three weeks afterwards, he returned to the same hospital, complaining that, in two days after his discharge, without any ostensible cause, he became affected with acute pain in the left flank, and also in the left side of the chest, attended with some fever, loss of appetite, and diarrhœa, but without cough or expectoration. Venesection did not relieve these symptoms, and when examined at the hospital, on the 28th of April, the following were the symptoms: the heat of surface was increased—pulse rather accelerated—thirst--white tongue—ten motions in the 24 hours—acute pain in the left flank, increased on pressure, and extending to the groin of that side—a tumour developed in the left hypogastric region, protruding several inches beyond the level of the false ribs. The inferior part of the left side of the chest sounded dull, and no respiration was there heard —but was clear in every other direction. The tumour appeared to M. Louis to be an enlarged spleen; and he thought this enlargement, as well as the diarrhœa, was owing to the suppression of the long-established drain from the ulcers on the legs. With this impression, M. Louis endeavoured to re-establish the said discharge by means of a large blister to the leg—and twenty leeches to the left flank and left side of the chest. In two days the pains had nearly ceased, and the diarrhœa was much diminished. The size of the spleen also decreased rapidly, and, by the 19th of May, there was no vestige of tumour in the left side, and the chest on that side was sonorous throughout. He was discharged cured. We forgot to mention that an issue had been established in the flank.

*Case* 2. This was a deaf person, but very intelligent, aged about 55 years, who came to the Hospital La Pitié in the beginning of last Winter, with a large ulcer, of several years' standing, on the malleolus internus. It was dressed regularly every day, and soon healed. In two or three days after the complete cicatrization, the patient, who had previously been in good health, lost his appetite, had nausea, malaise, lassitude, without any other symptom that could indicate any particular disease. The above continued to increase for eight days, when M. Recamier applied a large blister to the site of the healed ulcer. As soon as a suppurative discharge was established, the foregoing phenomena diminished, and, in the course of a week, they entirely disappeared. The patient remained three months in hospital, and had no return of complaint. The blister was then allowed to heal, and health continued.

*Case* 3. A female, aged 40 years, had a vaginal discharge for several years, which was suddenly and totally suppressed by a severe moral affliction. From that time the appetite and strength diminished—the patient complained of pains in the epigastrium—wasted in flesh—and, after three months, was obliged to enter the hospital. M. Louis endeavoured to re-establish the vaginal discharge by means of the vapour-bath —sinapisms, &c. In about eight days these remedies reproduced the vaginal discharge, from which period all the symptoms above mentioned gradually diminished, and at length disappeared.

*Case* 4. A female, aged 62 years, was received into hospital on the 23d of May of this year. The catamenia had ceased about 12 years previously, since which she was subject to palpitations of the heart and pains in her head. For these she had been bled eight times. Her legs swelled occasionally in the evenings. During the last six months she had leucorrhœa of inodorous character. Having been severely frightened by some ruffians one evening, and beaten by them, the discharge suddenly stopped, and soon after this she felt depressed, with head-ache, giddiness, some obscurity of vision, constant drowsiness, cramps in her limbs, with some dyspnœa and œdema of the feet. Nevertheless she was free from the palpitation; but her appetite disappeared. These symptoms continued unabated for eight days, and were in the same state when she entered the hospital on the 23d of May. She appeared to have a good constitution—pulse 84, regular, as were the motions of the heart, which did not appear enlarged—lungs sound. A large blister was applied to the inside of the right leg—vapour-bath to the lower half of the body—sinapisms to the feet. 24th. Pulse 96—the other symptoms the same. 20 leeches were applied to the lower extremities. 25th. During the bleeding of the leeches, some of the leucorrhœal discharge returned, but did not continue. The symptoms however, were all mitigated, and when the blister came to discharge freely, they disappeared, with the exception of considerable debility, which required tonics and nourishing diet. Some threatenings of her former symptoms, however, required the formation of an issue in the the thigh, which entirely removed them.

